# Social Discounting under Risk

**DOI:** 10.3389/fpsyg.2017.00392

**Published:** 2017-03-15

**Authors:** Jia Jin, Guanxiong Pei, Qingguo Ma

**Affiliations:** ^1^Business School, Ningbo UniversityNingbo, China; ^2^Academy of Neuroeconomics and Neuromanagement, Ningbo UniversityNingbo, China; ^3^School of Management, Zhejiang UniversityHangzhou, China; ^4^Institute of Neural Management Sciences, Zhejiang University of TechnologyHangzhou, China

**Keywords:** risk, prosocial behavior, generosity, social discounting, social distance

## Abstract

As a measure of how prosocial behavior depends on social distance, social discounting is defined as the decrease in generosity between the decision maker and the recipient as the social distance increases. While risk is a ubiquitous part of modern life, there is limited research on the relationship between risk and prosocial behavior. In the present experiment, we empirically test whether risk has an influence on social discounting. We use the choice titration procedure to examine this effect. Our data show that independent of risk, participants are less eager to forego money and exhibit more selfishness toward a specific person when the social distance increases; these findings are reflected in the hyperbolic model. Interestingly, risk influences the shape of the social discounting function, which is reflected in the notable different discount rates. Individuals who make decisions under risk yield a smaller discount rate than those who make decisions without risk, i.e., under risk subjects reduce less their generosity as a function of the social distance. Furthermore, this distinct type of generosity occurs typically among individuals with 10-distance recipients but not with the closest- and furthest-social-distance recipients.

## Introduction

Prosocial behavior is vital to social functioning. Many studies have shown that individuals take others’ interests into account and share resources in different social contexts (Twenge et al., 2007; Brañas-Garza et al., 2010; Corgnet et al., 2015; Padilla Walker et al., 2015; Simpson and Willer, 2015). It was demonstrated that social distance was one of determinants of prosocial giving (Brañas-Garza et al., 2010). Social discounting is a measure of how prosocial behavior depends on social distance, which is defined as the decrease in generosity between the decision maker and the recipient as the social distance increases (Jones and Rachlin, 2006, 2009; Jones, 2007; Takahashi, 2007, 2010; Brañas-Garza et al., 2010). In a modified dictator game, people always favored friends over strangers. Directed altruism increased giving to friends by 52% compared with random strangers (Leider et al., 2009). In another dictator game, even when the effect of reciprocity was controlled, donations to friends were still 35% higher than to strangers (Brañas-Garza et al., 2012). This means that our willingness to share goods and resources with other individuals is influenced by social distance. That is, we tend to be more generous with those whom we feel closer to than with those who are further from us in social distance.

The concept of social discounting, first suggested by Jones and Rachlin (2006), supposes that generosity decreases across social distance in a non-constant, hyperbolic way and is inspired by time discounting. It is described by the equation below:

$$\begin{array}{c} v=\frac{V}{1+kD} \end{array}$$

where *v* symbolizes the discounted value, which is the willingness to be generous toward a person at a given social distance, and *D* represents the social distance. The parameter *V* refers to the value of the undiscounted reward. The parameter *k* refers to the discount rate, i.e., the steepness and the asymmetry of the decline in generosity across social distance (Jones and Rachlin, 2006; Strombach et al., 2014; Ma et al., 2015). The hyperbolic discount function captures the trade-off between selfish and other-focused motives as a function of how much the decision maker cares about others.

In following studies, researchers also focused on the influential factors of social discounting like studies of time discounting. For instance, Rachlin and Jones (2008) confirm that the social discounting features a reversed amount effect when compared with the so-called amount effect for time discounting (devaluation of an amount of money with increasing receipt time delay). In time discounting, participants have a higher discount rate for smaller amounts of money, while in social discounting, participants have a higher discount rate for larger amounts of money. In addition to the magnitude of the reward, other influential factors, such as the decision-making environment and contextual differences (Jones, 2007), whether the participant smokes and how often (Jones, 2007), the cultural background of the decision maker (Ito et al., 2011; Strombach et al., 2014; Ma et al., 2015), anonymity (Locey and Rachlin, 2015), and so on, have been studied. Strombach et al. (2015) also studied the neural basis of social discounting through the use of neuroimaging tools and developed a biological model with the interplay of two brain structures-the ventromedial prefrontal cortex and the temporoparietal junction. The neural perspective opens up new avenues for addressing social issues. As a newly emerging field, the inner mechanisms and other influential factors of social discounting need to be explored further.

Because decision-making in daily life always involves risk components, the hypothesis of certainty concerning prosociality is impractical and not representative of the real world. To understand more about social discounting in the real economic world, we intend to investigate whether and how risk will influence social discounting behavior in this study. There are two reasons for this:

First, there are limited studies concerning the relationship between risk and prosocial behavior. Stellar et al. (2012) proposed that individuals who were more charitable, empathic, and generous toward others affiliated with social groups to reduce uncertainty and risk. Kovářík and Van der Leij (2014) firstly studied the relationship between risk aversion and social network structure. It was found that locally superior information on benefits made risk-averse individuals more likely to link to friends of friends. Angerer et al. (2014) demonstrated that risk was closely related to prosocial behavior, but the relationship between risk preference and reciprocal altruism has not been clearly studied or explained. Rand and Epstein (2014) discussed altruism under extremely risky and costly conditions; however, the conclusion is difficult to generalize. Further studies are needed. Second, social discounting may be deeply influenced by risk. It was found that individuals were inclined to employ the tend-and-befriend strategy under conditions of risk and uncertainty (Stellar et al., 2012). The tend-and-befriend reaction theorizes that increasing the needs of others results in an increase in prosocial behaviors (Taylor et al., 2000; Pickett and Gardner, 2005; Taylor, 2006). It means that people tend to be more generous under risk to search for support. Consistent with the tend-and-befriend theory, a recent meta-analysis on risk preferences in choices for self and others demonstrated that choices for others were significantly more risk averse when decision makers were reciprocally related to recipients (Atanasov, 2015). For relationship preservation, they tended to minimize anticipated blame from losses and tried to maximize credit for gains. When faced with risk, they would maximize the recipient’s utility and be more generous to recipients to preserve the beneficiary relationship (Atanasov, 2015). In a multilateral cooperation game, two different type of costly punishment were identified: punishment of free-riders by cooperators (to avoid the transgressions of moral standards) and free-riders’ punishment of other free-riders (to avoid the risk of losing the competitive advantage). Although the psychological underpinnings were different, participants tended to enforce cooperation when faced with risk (Espín et al., 2012). Based on these theories above (Taylor et al., 2000; Espín et al., 2012; Stellar et al., 2012; Atanasov, 2015), we speculate that participants would be more generous under risk condition.

In the present experiment, we empirically test the influence of risk on social discounting. Referring to previous studies (Jones and Rachlin, 2006; Strombach et al., 2014), we have designed the experiment using the choice titration procedure. Participants are faced with two conditions – with risk and without risk. First, we hypothesize that generosity declines as a function of social distance independent of risk and has a good fit with the hyperbolic model. Second, we speculate that risk influences the shape of the social discounting function, which is reflected in the different discount rates.

## Materials and Methods

### Participants

All participants have been recruited from Zhejiang University and are healthy, native Chinese speakers. The sample consists of *N* = 65 (38 men) with an average age of 21.15 years (*SD* = 2.17). All participants are recruited using an internal bulletin board system. Due to their incomplete comprehension of the experiment, data from six subjects (two male) have been eliminated, leaving 59 valid participants for final data analysis. The study has obtained ethics approval from the Internal Review Board of Zhejiang University. The privacy and rights of the participants are protected. Each participant has given informed consent before taking part in the study. All participants received monetary compensation.

### Experiment Design and Procedure

The current study empirically adapts a social decision-making task originally developed by Jones and Rachlin (2006) to investigate the influence of risk on social discounting. In the experiment, each participant completes a series of choices with or without risk. All trials are randomly presented.

As in Jones and Rachlin’s (2006), decisions are made for the following seven social distances: 1, 2, 5, 10, 20, 50, and 100. Social distance is measured on a ratio scale and converted to a scale consisting of 100 icons. The first icon on the left side represents the participant while the other icons represent other people in the participant’s social environment. The second icon (social distance 1) represents the person within the participant’s social environment to whom the participant is closest, e.g., his/her mother. The icon on the opposite end of the scale (social distance 100) represents a person who is the farthest from the participant socially, a person for which the participant has neither positive nor negative feelings – e.g., a random stranger.

**FIGURE 1:**
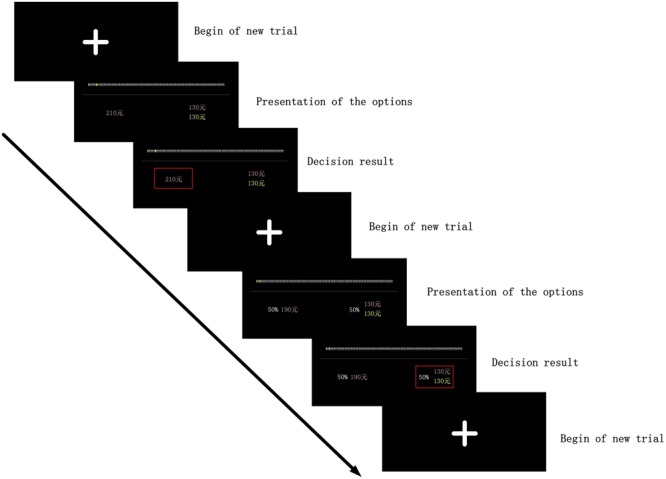
**Example of the social discounting experiment in non-risk and risk condition.** In each trial, there are three screens. The first screen notices that the trial begins. The second screen shows the two options, in which the left option means a selfish choice and the right option means a generous choice. The yellow-coded numbers under the scale indicate the recipient and the magnitude of his/her reward while the pink-coded numbers indicate the participant himself/herself. The third screen shows the decision result.

In each trial, the participant has to select between a selfish and a generous option that yields either a large reward for only the participant or a smaller reward for the participant and a reward for the recipient. The recipient is the person at a given social distance, denoted as yellow on the ratio scale. Each participant is asked to imagine the recipient as a real person at this specific social distance. The yellow-coded numbers under the scale in **Figure 1** indicate the recipient and the magnitude of that reward. The selfish option changes across trial repetitions in increments of 20 Yuan from 130 to 290, while the generous option has a fixed magnitude of 130 Yuan for the participant and the recipient, respectively. In trials with risk, the social discounting part is similar to the procedures originally adopted by Jones and Rachlin, with the added element of risk. Therefore, each participant is required to participate in 126 trials (2 conditions: risk and non-risk × 7 types of social distance × 9 monetary amounts for the selfish option). We use the risk trial shown in **Figure 1** as an example. The social distance in this trial is 2. Choosing the left option means the participant receives 190 Yuan with a 50% risk, while choosing the right one means the participant and the recipient each receive 130 Yuan with a 50% risk. In other words, there is a 50% probability that both will receive the reward, and an equal probability that they will receive nothing. Trials without risk share the same experimental process as those under risk, only the choices involved no risk. So the payments chosen would be implemented for sure. The experiment is presented using E-Prime 2.0 software package (Psychology Software Tools, Pittsburgh, PA, USA).

In the subsequent task, participants are required to complete a questionnaire naming and depicting his or her relationships to the recipients that are representatives for each of the seven social distances, along with such personal information as sex and age. Participants receive a participation fee of 10 Yuan. At the end of each experiment, one trial within the experiment is randomly chosen, and the participants receive another payment of 10% of the real decision value. That is, if a selfish option is selected under a non-risk condition, the participant receives between 16.5 Yuan and 24.5 Yuan, depending on the trial chosen. If the decision is generous, the participant receives 16.5 Yuan, and the other person receives 6.5 Yuan. If the chosen trial is conducted under a condition of risk, the participant tosses a coin to determine whether he/she will receive the money. This payment rule was informed to the participants before the formal experiment started.

Participants are asked to record the address of the virtual interaction partner in that trial. The interaction partners receive their rewards by mail. For participants who didn’t know the complete address details or the bank account of the virtual interaction partner or chose a trial in which the virtual interaction partner was a stranger, they had the option to donate their interaction partner’s money to a charity (China Youth Development Foundation). Information about this option is only given at the end; thus, it does not interfere with the participants’ choices. The experiment is performed in an incentive-compatible way and does not involve deception; thus, it meets the standards for economic research (Bonetti, 1998; Schram, 2005).

### Data Analysis

**FIGURE 2:**
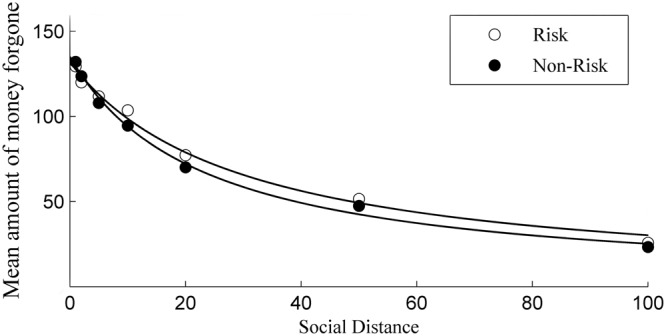
**Fitting of the hyperbolic discounting function for risk and non-risk conditions**.

In the social discounting test, at each level of social distance, we got participants’ selfish or generous choices at nine sequential selfish options, with increments of 20 Yuan from 130 to 290. Therefore, firstly, we calculated the cross point between the selfish and generous choices at which the participant evaluated the two choices indifferently by titrating the selfish reward magnitude. Logistic regression was used to determine the cross point, i.e., the point at which the statistical probability of answering selfishly and generously is 50%. If a participant always decides selfishly or generously at a particular social distance level, the intersection points are assumed to be 120 and 300 Yuan, respectively. We got seven cross points for each participants since there is seven social distance in our experiment. The cross point shows how much money the participant is willing to sacrifice to give a reward to the specific person with different social distance (Jones and Rachlin, 2006). Secondly, we calculated the amount of money forgone at each social distance for each participant. The amount of money forgo is equivalent to the cross point minus 130, the quantity of money the subject receives if he/she chooses the generous option. Thirdly, we used the seven pairs of amount of money forgo and social distance to estimate the undiscounted value *V* and discount rate *k* by the above mentioned standard hyperbolic model for each participant in the two conditions. The average amount of money forgo was also calculated to estimated the general *V* and *k* for the two conditions. The fitted curve was show in **Figure 2**. Finally, to examine if there was significant differences between risk and non-risk conditions, the two-related sample non-parametric Wilcoxon test was conducted for discount rate *k*, as well as the amount of money forgone at each social distance.

## Results

Results show that participants from both risk and non-risk conditions are less eager to forego a reward by being generous as the social distance increases, which replicates previous research findings (Jones and Rachlin, 2006; Jones, 2007; Rachlin and Jones, 2008). A standard hyperbolic model (Mazur, 1987) is matched to the mean value of the amount forgone separately for each condition, indicating a good fit for the risk (*X*^2^ = 0.6177, *R*^2^_corr_ = 0.9925) and non-risk data (*X*^2^ = 0.4552, *R*^2^_corr_ = 0.9949), which is summarized in **Table 1**. **Figure 2** presents the mean amount forgone and the hyperbolic fitted curve of the two conditions.

**Table 1:** The estimation result of risk and non-risk conditions.

Model	Condition	Mean fit	Fitted parameters
Hyperbolic model	Risk	*r*^2^ = 0.9925; *X*^2^= 0.6177	*k* = 0.03361; *V* = 131.9131
Hyperbolic model	Non-risk	*r*^2^ = 0.9949; *X*^2^ = 0.4552	*k* = 0.04355; *V* = 135.0593

*The standard hyperbolic model was used to estimate data of the two conditions separately. The estimated results of two free parameters *k* and *V*, as well as Goodness of Fit, are shown. Both the data sets fit the model well and the risk condition has smaller discounting rate than that of non-risk condition.*

The discount function has two free parameters, *V* and *k*. *V* symbolizes the undiscounted value, and *k* refers to the individual discount rate (Green and Myerson, 2004; Jones and Rachlin, 2006). We find significant differences in *k* across the two conditions (*Z* = -2.573, *p* = 0.010); the subjects with the non-risk condition (mean value: 0.1052) have larger *k*-values than those with the risk condition (mean value: 0.0166). Thus, in the risk condition, participants have a smaller discount rate than those of non-risk condition, which indicates that generosity levels decay at a slower rate across social distance.

**FIGURE 3:**
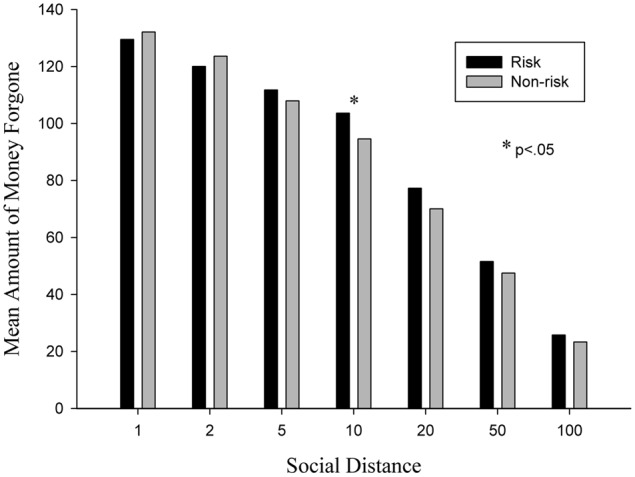
**Mean amount of money forgone per social distance in risk and non-risk conditions**.

Moreover, we also examine the effects of risk when comparing levels of generosity per social distance. Because heterogeneous variations exist, several Wilcoxon tests have been conducted. As summarized in **Table 2** and **Figure 3**, there are significant differences between the two conditions at social distance 10 (*Z* = -2.143, *p* = 0.032). Compared to the non-risk (mean value: 94.57) condition, the risk condition (mean value: 103.61) has greater mean amounts of forgone money.

**Table 2:** The statistical results for each social distance between risk (*R*) and non-risk (*N*) conditions.

Social distance	*z*-Value	*p*-Value	Mean	Standard deviation	Mean rank
1	-0.779^a^	0.436	*R* = 129.49; *N* = 132.10	*R* = 24.745; *N* = 25.547	*R* = 11.21; *N* = 14.65
2	-1.371^a^	0.170	*R* = 120.03; *N* = 123.63	*R* = 37.329; *N* = 39.944	*R* = 15.25; *N* = 12.72
5	-0.076^b^	0.939	*R* = 111.75; *N* = 107.92	*R* = 33.238; *N* = 37.928	*R* = 16.75; *N* = 16.25
10	-2.143^b^	0.032	*R* = 103.61; *N* = 94.57	*R* = 35.749; *N* = 44.571	*R* = 19.16; *N* = 14.46
20	-0.109^b^	0.913	*R* = 77.28; *N* = 70.08	*R* = 37.848; *N* = 45.492	*R* = 21.16; *N* = 19.90
50	-0.337^b^	0.736	*R* = 51.57; *N* = 47.52	*R* = 41.952; *N* = 36.964	*R* = 20.78; *N* = 22.37
100	-0.407^b^	0.684	*R* = 25.78; *N* = 23.33	*R* = 36.447; *N* = 38.421	*R* = 15.81; *N* = 12.32

*The two-related sample non-parametric Wilcoxon test results of *p*-value, *Z* score, mean rank, mean amount of money forgo and its standard deviation of the two conditions at each social distance were shown. There was significant difference of amount of money forgo at social distance 10. Participants were more generous under risk condition (higher amount of money forgo). ^a^Based on positive ranks. ^b^Based on negative ranks.*

## Discussion

The objective of this study is to investigate the effect of risk on social discounting. We employ the decision-making experiment using the choice titration procedure to study differences in social-distance-dependent prosocial behavior between risk and non-risk conditions. The overall results support the discovery of (Jones and Rachlin, 2006; Rachlin and Jones, 2008). That is, the closer our interaction recipients are to us, the more likely we are to be generous. The discounting results of both conditions can be depicted by a standard hyperbolic function. Further analyses demonstrate that risk seems to have an effect on distance-dependent levels of generosity, which is reflected by the lower degree of discounting for the risk condition. From the perspective of the tend-and-befriend theory and the risk preference theory (Taylor et al., 2000; Stellar et al., 2012; Atanasov, 2015), individuals tend to be more generous under risk to search for support and maximize the recipient’s utility to preserve the beneficiary relationship. It means that a smaller discount rate should be yielded under risk condition. Our results support this hypothesis.

It was demonstrated that social distance was one of the determinants of prosocial giving (Brañas-Garza et al., 2010). By comparing the distinction between the two conditions in each social distance, we find significant differences in generosity levels at social distance 10. However, for recipients who were closest to and furthest from the participants, the money forgone does not change much when there is a 50% probability of getting nothing.

For recipients who are at the closest social distance, there is a fixed tendency to share and a high level of willingness to make sacrifices (Ostaszewski and Osiński, 2015). Participants may perceive their interests as almost aligned with the recipients’ benefits. Thus, participants tend to be generous and exhibit a kind of attitude-behavior consistency. In Chinese culture, people are typically family-oriented and collectivistic. Even when a person becomes an adult and is economically independent, he is still subject to the influence of his parents and exhibits filial piety and submission. The younger ones have the responsibility of caring for their elderly parents (Ma et al., 2015). This special family-child relationship, based on Confucianism, still deeply affects the Chinese peoples’ decision-making with regard to sharing among their closest social relationships. So the external risk does not influence individuals’ generosity to people with the closest social distance, especially in China.

For recipients who are at social distance 10, previous studies show that individuals tended to employ the tend-and-befriend strategy and maximize the recipient’s utility under conditions of risk and uncertainty (Taylor et al., 2000; Stellar et al., 2012; Atanasov, 2015). While risk is a trigger of attachment-related prosocial behaviors in humans (Mikulincer et al., 2006), it needs preconditioning; consequently, the decision maker’s social closeness to the recipient is a key factor in determining how risk affects altruism. As shown in the previous research, people always favored friends and tended to give much more to friends than to random strangers (Leider et al., 2009; Brañas-Garza et al., 2012). Individuals would be more generous to recipients only when they need to preserve the beneficiary or reciprocal relationship (Atanasov, 2015). In this study, participants may have a relatively high degree of trust with recipients at 10-distance and expect to receive a return or support to provide mutual protection. That may be the reason why the amount of money forgone is significantly higher in a risk condition at 10-distance. It means we search for support and are more generous with those from whom we expect to provide support; we do not indiscriminately befriend anyone (Margittai et al., 2015).

The tend-and-befriend theory and risk preference theory also provide an explanation for instances where there is no difference in generosity between two conditions when faced with the most distant recipients. We are more generous with people at close social distance who are more likely to provide us with support and comfort, but not with strangers. In other words, we don’t have the motivation to preserve the relationship which is not reciprocal (Taylor et al., 2000; Stellar et al., 2012; Atanasov, 2015). From another prospect, Strombach et al. (2015) shows that the temporoparietal junction is associated with overcoming egoism bias in social discounting. In the most distant condition between the participant and beneficiary, greater effort should be exerted to overcome the temptation to be selfish. Thus, when faced with a total stranger or someone they do not know well, participants tend to be selfish. So it is not surprising to find that insignificant differences in generosity levels between the two conditions exist at the largest social distance.

The present study has two major limitations. To begin with, in the risk condition, the subjects were betting over one half of the money (in expected terms) compared to the non-risk condition. The influence of risk on social discounting might be interfered by the well-known reverse amount effect. To be specific, from the perspective of expected value, the probability information in the risky setting may be combined with the amount of the reward. For example, ‘50% chance to receive ¥130 alone’ may be simplified to ‘receive ¥65 alone.’ That means that adding the external risk to both alternatives is equal to proportionally diminishing the amount of the rewards. In previous studies, a reverse amount effect has been found in social discounting. Lower amounts of money are discounted less steeply by social distance than higher amounts (Jones, 2007; Rachlin and Jones, 2008; Charlton et al., 2012). Thus, decreasing the amount of the rewards can also decrease the degree of social discounting. For future research, the stakes in the risk condition should be doubled and the differences in the levels of social discounting across conditions can be studied free of reverse amount effects. Another limitation of this study is that there was only one level of risk was measured in this study. For future research, the risk level can be altered. A probability of more or less than 50% may have a different impact on social discounting, which will be helpful in gaining a deeper understanding of altruistic decision-making under risk.

## Conclusion

Our study investigates risk-specific differences in social-distance-dependent levels of generosity. Participants in both risk and non-risk conditions are willing to forgo a quantity of money for the benefit of others, and the generosity levels decrease with increases in social distance, which is depicted by hyperbolic function. The analysis further reveals that risk influences the shape of the social discounting function. The individuals under risk yield a smaller discount rate than those who are not under risk. Furthermore, this distinct type of generosity occurs most notably among individuals with 10-distance recipients; it does not occur among the closest- and furthest-social-distance recipients.

## Author Contributions

JJ made substantial contributions to the conception of the work, the acquisition, analysis and interpretation of data, as well as drafting and revising the manuscript. GP made substantial contributions to the conception of the work, as well as the interpretation of data. QM made substantial contributions to the conception of the work, as well as the analysis and interpretation of data and in revising the manuscript. All authors gave approval of the final version.

## Conflict of Interest Statement

The authors declare that the research was conducted in the absence of any commercial or financial relationships that could be construed as a potential conflict of interest.
